# Advances in Colorimetric Strategies for Mycotoxins Detection: Toward Rapid Industrial Monitoring

**DOI:** 10.3390/toxins13010013

**Published:** 2020-12-24

**Authors:** Marjan Majdinasab, Sondes Ben Aissa, Jean Louis Marty

**Affiliations:** 1 Department of Food Science & Technology, School of Agriculture, Shiraz University, Shiraz 71441-65186, Iran; majdinasab@shirazu.ac.ir; 2 BAE-LBBM Laboratory, University of Perpignan via Domitia, 52 Avenue Paul Alduy, CEDEX 9, 66860 Perpignan, France; sondes.benaissa@univ-perp.fr

**Keywords:** mycotoxins, colorimetric detection, rapid tests, ELISA, lateral flow assays, microfluidics, nano-materials, food safety, commercialization

## Abstract

Mycotoxins contamination is a global public health concern. Therefore, highly sensitive and selective techniques are needed for their on-site monitoring. Several approaches are conceivable for mycotoxins analysis, among which colorimetric methods are the most attractive for commercialization purposes thanks to their visual read-out, easy operation, cost-effectiveness, and rapid response. This review covers the latest achievements in the last five years for the development of colorimetric methods specific to mycotoxins analysis, with a particular emphasis on their potential for large-scale applications in food industries. Gathering all types of (bio)receptors, main colorimetric methods are critically discussed, including enzyme-linked assays, lateral flow-assays, microfluidic devices, and homogenous in-solution strategies. This special focus on colorimetry as a versatile transduction method for mycotoxins analysis is comprehensively reviewed for the first time.

## 1. Introduction

Widespread mycotoxins contamination of food and feed poses a serious menace for human’s health and contributes to massive economic losses in the agriculture industry. Mycotoxins are chemically diverse groups of low molecular weight fungal metabolites that are almost unpredictable and unavoidable in crops and have a wide variety of toxic effects [1]. These thermal-stable fungal toxins affect a broad range of agricultural products including cereals, cereal-based foods, dried fruits, wine, milk, coffee beans, cocoa bakery, and meat products [2]. 

Hitherto, over 300 kinds of mycotoxins have been characterized, but only about a dozen have led the priority list of risk assessment due to their high occurrence in food staples and severe health effects [3]. Representative mycotoxins include aflatoxins (AFs), ochratoxins (OTA), fumonisins (FB), zearalenone (ZEN), patulin (PAT), deoxynivalenol (DON), and trichothecenes. According to the International Agency for Research on Cancer (IARC), some are proved to be strong carcinogenic agents such as aflatoxin B1 (AFB1) while others are under suspicion to have carcinogenic effects [4]. Nonetheless, all of them have shown acute and chronic toxicities [5]. Hence, stringent regulations relating to mycotoxins have been established in many countries to protect the consumer from their harmful effects [6]. The established maximum limits (MLs) differ depending on the mycotoxin and the targeted foodstuff. In particular, the strictest regulations have been set for aflatoxins in the processed food products for infants [2]. In addition to the regulatory framework, consumers have become recently more aware of health and food quality. Therefore, research on the development of high-throughput, real-time, and reliable portable detection methods for food safety augmented [7].

The operation procedure should be simplified continuously for users’ convenience, avoiding the need for laboratory-based techniques. Many instrumental methods have been used from the very early discovery of mycotoxins till now, such as thin-layer chromatography (TLC), high-performance liquid chromatography (HPLC), in combination with different detectors (e.g., fluorescence, diode array, UV), liquid chromatography coupled with mass spectrometry (LC-MS), liquid chromatography-tandem mass spectrometry (LC-MS/MS) and gas chromatography-tandem mass spectrometry (GC-MS/MS) for mycotoxin analysis [2]. Owing to their high sensitivity and precise analysis, such techniques present the gold methods to control mycotoxins levels in food samples in compliance with the regulatory framework. Reviews of these methods have been summarized and published elsewhere [8,9]. Despite their analytical merits, chromatographic methods involve tedious multistep processes that are time-consuming and require highly skilled personnel. Moreover, expensive and bulky instruments restrict their use for in-situ mycotoxin analysis. Therefore, more convenient and user-friendly methods were still highly desirable for the rapid monitoring of mycotoxins’ traces in food and feed.

Consequently, optical methods have received great attentions in developing rapid detection kits specific to common mycotoxins. Among different sensing strategies, colorimetric detection methods are particularly well-suited for on-site biosensing due to their simple readout and operation. They can serve for qualitative, semi-quantitative or quantitative methods for a rapid screening in field, in silo, or during the agri-food processing. The portability of such miniaturized tools is profitable for industrials to validate their products’ conformity in accordance with regulatory limits. 

Colorimetric methods can be classified based on the type of color-generating probes (dyes, enzymes, nanomaterials) and the sensing reaction phase (solution-based and solid substrate-based). Enzyme-linked immunosorbent assays (ELISA) are the most popular colorimetric screening tools that reached successfully the commercialization stage for mycotoxins analysis along with some lateral flow immunoassays (LFIA). Thanks to their unique features, detection kits relying on these two techniques are being manufactured by multiple companies worldwide. Despite the current market competitiveness, the colorimetric methods dedicated to the determination of representative mycotoxins in foodstuffs continue to attract industrials for a reliable and cost-effective monitoring. Campbell et al. have recently reviewed the available commercial kits [10], emphasizing that antibody-based schemes conquer the most part of the market owing to their superior specificity for real-world applications.

However, many successful proofs of concept were described in the recent literature using either chemical sensing or other bioreceptors. In particular, aptamers are short single-stranded oligonucleotides (DNA or RNA) which can replace antibodies as recognition element in sensing strategies. They exhibit several advantages such as high stability, low production cost, high affinity and specificity, and high sensitivity. Parallelly, emerging nanomaterials had led to an unprecedented improvement of these alternative sensing strategies in food safety control. 

Overall, the rapid development of colorimetric methods has brought many opportunities for rapid mycotoxins detection. Many emerging and novel (bio)assays have been reported as competitive analytical tools with easy operation and fast visible response. However, to date, there are very few reviews that focused on the colorimetric transduction application for mycotoxins analysis in food matrices regardless the bioreceptor nature and the target mycotoxin type. Thus, it is necessary to give a comprehensive summarization. This helps to understand the current trends and assist decision-makers to apply such cost-effective technologies in agri-food industries. The aim of the present review is to place the diverse colorimetric methods (solution-based (bio)assays, ELISA, lateral flow assays, microfluidics) within a critical framework that compares the merits and limitations of each methodology and highlights the progress that has been made in recent five years. Current figures of merit of rapid colorimetric methods with great potential for industrial applications are thoroughly discussed.

## 2. Common Colorimetric Probes

### 2.1. Enzymes-Based Probes

Enzymes are robust signal amplification systems in bioassays and biosensors. They can be used in both optical and electrochemical sensing strategies. The principle of the enzyme-based colorimetric assays is to detect target analyte through the enzymatic conversion of a chromogenic substrate into a colored product. The produced color can be detected by the naked eye (qualitative methods) and through spectrophotometry or colorimetric analysis software (semi-quantitative or quantitative methods). The three most common types of enzyme-based colorimetric probes—including enzyme horseradish peroxidase (HRP), G-quadruplex sequences or DNAzymes, and acetylcholinesterase (AChE)—have been applied for colorimetric detection of mycotoxins and are hence discussed here.

**Figure 1:**
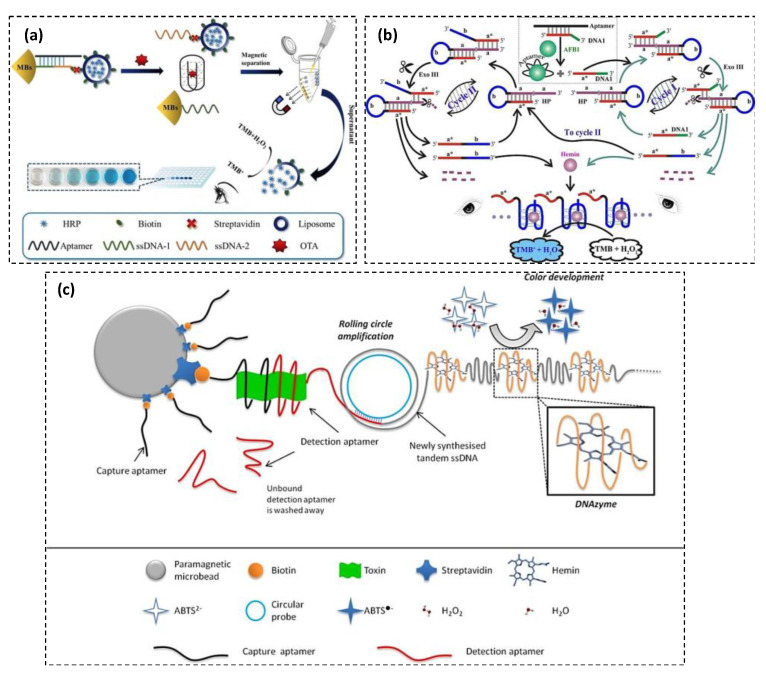
(**a**) Schematic illustration of the colorimetric aptasensor for OTA detection based on HRP-encapsulated liposome; (**b**) developed aptasensor for AFB1 detection using G-quadruplex as the signal reporter. Domain a is complementary to domain a*. Domain b is the caged G-rich sequence. Exo III performs the cyclic cleavage reactions in Cycles I and II; (**c**) aptasensor for OTA detection, based on rolling circle amplification and an auto-catalytic DNAzyme structure. Reproduced with permission from [11,15,16], respectively.

HRP, found in the roots of horseradish plant, is the most popular enzymatic marker in bioassays due to ability to be conjugated with antibodies or other recognition elements, while preserving its activity, low-cost, and versatility. HRP can catalyze the reaction of hydrogen peroxide with certain organic, electron-donating substrates to yield highly colored products. An extensive range of electron-donating dye substrates are commercially available for use as HRP detection reagents. Some of them can be employed to form soluble colored products suitable for use in spectrophotometric detection methods, while other substrates form insoluble products that are mainly appropriate for staining techniques. Among them, 3,3′,5,5′-Tetramethylbenzidine or TMB is widely used as a soluble chromogenic substrate for colorimetric detection in enzyme-linked immunosorbent assay (ELISA) and other bioassays. However, some HRP-based bioassays suffer from limited sensitivity due to small amount of enzyme (i.e., HRP) that catalyzes chromogenic substrate. To address this issue, Lin et al. presented a method to combine the analyte-recognition element complex with a large number of enzymes [11]. They developed a liposome-based colorimetric aptasensor for ochratoxin A (OTA) detection in a TMB-H_2_O_2_ reaction medium. In this context, liposome as a sphere-shaped vesicle with hydrophobic and hydrophilic character was used for encapsulation of HRP. The main component of the detection system was a dumbbell-shaped probe including magnetic beads (MBs), double-stranded DNA (dsDNA), and HRP-encapsulated liposome (Figure 1a). The dsDNA was formed by the hybridization between OTA aptamer and its complementary probes ssDNA-1 and ssDNA-2. ssDNA-2 was conjugated with liposome and used as detection probe. In the presence of OTA, the aptamer combined with OTA to form G-quadruplex, resulting in the release of the ssDNA-2 and the HRP-encapsulated liposome. Each liposome containing a large quantity of HRP was lysed by adding the mixed solution of TMB and H_2_O_2_. HRP catalyzed H_2_O_2_-mediated oxidation of TMB and resulted in color change from colorless to blue. The assay was highly sensitive due to the signal amplification caused by the large amount of HRP embedded in liposome. The limit of detection (LOD) was obtained 0.023 ng·mL^−1^. The assay was simple, low-cost, highly selective and reliable for the analysis of real samples. However, the reaction time for the G-quadruplex formation (40 min) and TMB oxidation (20 min) was too long. The aptasensor was also applied for OTA detection in corn samples.

Haem peroxidases such as HRP use protein scaffolds that activate heme to react with H_2_O_2_ [12]. There is extensive information on the reaction mechanism and properties of protein-based peroxidases. It was recently revealed that certain nucleic acid sequences have the ability to catalyze reactions similar to those carried out by heme. These nucleic acid sequences are non-canonical Guanine-rich structures with stacked G-tetrads assembled by Hoogsteen hydrogen-bonding. These sequences, named as G-quadruplex (G4), are able to bind hemin (iron (III)-protoporphyrin IX) to form a unique type of G4 DNAzyme or RNAzyme with powerful peroxidase-mimicking activity [13]. In comparison with natural protein peroxidases, G4 DNAzymes/RNAzymes show several advantages such as small size, easy synthesis, more stability, and facile manipulation, which make them good candidates in biosensing [13]. However, they suffer from relatively low catalytic activity compared to protein peroxidases which restricts their further development and application [14]. To overcome this limitation, several strategies have been developed to improve the catalytic efficiency of G4 DNAzymes/RNAzymes. These include (1) addition of polycationic amines such as spermine, spermidine, and putrescine; (2) addition of the nucleotide ATP to DNAzyme reactions; (3) conjugation of hemin with the G4-quadruplex moiety through covalent linkage or with cationic peptides; and (4) flanking adenine or cytosine nucleotides on G-quadruplex activities [12]. Incorporation of aptamers and DNAzymes as functional nucleic acids results in simple detection of target analyte by visual color development.

Using G-quadruplex as the signal reporter, a colorimetric aptasensor was developed for AFB1 detection [15]. The aptasensor was fabricated by the combination of an ingenious hairpin DNA probe with exonuclease III (Exo III)-assisted signal amplification. The hairpin DNA probe contained a 3′-protruding segment (domain a) as the recognition unit, the stem zone (domains a and a*), and a caged G-rich sequence located in the loop region (domain b). The presence of the AFB1 activated the continuous cleavage reactions by Exo III toward a hairpin probe, resulting in the autonomous accumulation of numerous free G-quadruplex sequences, which catalyzed the oxidation of TMB by H_2_O_2_ to generate a colorimetric signal (Figure 1b). The aptasensor represented many advantages including high sensitivity (LOD of 1 pM), good selectivity, simple operation, wash-free, label-free format, low-cost, naked-eye detection, and applicability to samples with complex matrices. However, the assay time was long (incubation time 40 min). The assay was used for AFB1 detection in peanut samples.

Detection with the aid of magnetic beads-based separation has emerged as a rapid, simple, reliable, and efficient alternative to conventional immobilization methods. In this regard, a colorimetric aptasensor based on apta-magnetic separation assisted with DNAzyme was developed for AFB1 detection [17]. The procedure consisted of one-step separation of AFB1 by biotinylated aptamer conjugated to streptavidin magnetic beads which was followed by the addition of DNAzyme modified aptamer in the presence hemin and TMB/H_2_O_2_ to produce a colorimetric signal. The aptasensor was able to detect as low as 40 ppb and 22.6 ppb OTA visually and by spectrophotometer, respectively. The developed assay was selective, reliable, inexpensive, and rapid (incubation time 15 min). However, the incubation time of DNAzyme was long (30 min). The aptasensor was able to detect AFB1 in food samples.

Sensitivity of DNA-based biosensors can be significantly increased using a technique known as rolling circle amplification (RCA). RCA is an isothermal enzymatic amplification process of DNA where a short DNA or RNA primer is amplified using circular DNA template. With proper application of this technique, it is possible to synthesize large quantities of any type of nucleic acid strand. In this context, a highly sensitive aptasensor based on RCA and an auto-catalytic DNAzyme structure was designed for OTA detection [16]. In this work, a capture aptamer was linked to paramagnetic beads for specific capturing of OTA while a second aptamer was applied for OTA detection. The detection aptamer contained a DNAzyme producing sequence and an RCA priming sequence for the isothermal DNA amplification triggered by a circular ssDNA. When OTA was captured, the circular DNA was amplified, generating a single-stranded and tandem repeated long homologous copy of its sequence. In the DNA strand, a self-catalytic structure was formed with hemin as the catalytic core causing a blue color in the presence of 2,2′-azino-bis(3-ethylbenzothiazoline-6-sulfonic acid) (ABTS) and H_2_O_2_ (Figure 1c). A low LOD of 1.09 × 10^−12^ ng·mL^−1^ was obtained. Although the aptasensor was highly sensitive and selective, it suffered from long incubation time and complicated operation with multiple steps of washing and separation. The aptasensor was used for OTA detection in urine samples.

There are various enzymes—such as cholinesterase, urease, glucose oxidase, etc.—which have been employed in mycotoxin detection methods based on enzymatic inhibition. AChE (obtained from electric eel) is the most commonly used enzyme due to its susceptibility toward mycotoxin [7]. It can be used for the detection of aflatoxin B1 (AFB1) due to the inhibitory effect of AFB1 to AChE enzymatic activity [18]. It has been proven that AChE is inhibited by the AFB1 due to non-covalently binding of toxin at the external site, which is placed on the active site gorge entrance (located at the tryptophan residue) [7]. Based on AChE inhibition, AFB1 was determined by a colorimetric method (Ellman’s method) developed on chromatography paper [19]. In this work, genipin cross-linked chitosan was used for AChE immobilization. For the colorimetric detection of AFB1 on microfluidic paper-based analytical device (µPAD), AChE immobilized on cross-linked chitosan was loaded on the edges of the flower-shaped µPAD. Then, AFB1 solution and 5,5-dithiobis-2-nitrobenzoic acid (DTNB, Ell-man’s reagent) solution were applied at the center of flower-shaped µPAD. After 3-min incubation, acetylthiocholine iodide (ATCh) solution was also added at the center, and incubated for 5 min. The Ellman’s colorimetric assay is based on the reaction of thiocholine (a product of enzymatic hydrolysis of ATCh) with DTNB to form a colored product. In the presence of AFB1, the AChE activity on ATCh substrate is inhibited resulting in failure to form a colored product. Cross-linking of chitosan resulted in a colorimetric signal enhancement. The assay was simple, low-cost, rapid (detection time ≈ 8 min), and fairly selective. However, the sensitivity of the assay was not reported. The assay was used for the detection of AFB1 in spiked corn samples.

AChE is considered very stable but lack of selectivity towards many toxins such as carbamates, organophosphate pesticides, anatoxin-a (a natural neurotoxic), and mycotoxins, which restrict its applicability. To address this issue, many efforts have been made to produce mutants of AChEs to improve the selectivity of enzyme against a specific toxin. Genetic modification of enzyme can also improve its stability and the assay sensitivity [20].

Representative examples of recent developed enzyme-based probes for the colorimetric detection of mycotoxins are reported in Table 1.

### 2.2. Nanomaterial-Based Probes

Accelerated by the advances in nanomaterials (NMs), colorimetric methods for the detection of mycotoxins have undergone a rapidly developing stage in the past few years [22]. Their nanometric size (less than 100 nm) and unique physicochemical features, including distinctive optical and catalytic properties, have promoted the extensive use of nanostructured materials in colorimetric methods. Accordingly, researchers handled each nanomaterial differently to adapt it with the desired function in the sensing assay. It is widely reported that NMs are attractive candidates to immobilize bioreceptors, including enzymes, antibodies, and aptamers, thanks to their large size to volume ratio, which provides a high specific active surface. In particular, magnetic nanoparticles (MNPs) from iron-based nanoparticles are widely used in colorimetric bioassays to capture, separate, and enrich target analytes, especially when a low detection limit is required. However, we emphasize in this section the signaling roles of NMs in colorimetric methods dedicated to mycotoxins detection. Glimpsing at the relevant literature, two prominent roles of NMs are depicted. NMs mainly based on metal nanoparticles show color switching tunable properties and are thereby used as direct colorimetric probes. Enzyme-like NMs (or nanozymes) also contribute to the advances in colorimetric assays, particularly through peroxidase and oxidase-like catalysis that generate colored products. Some NMs can be also employed as signal mediators to enhance assay sensitivities in cascade amplification systems.

As optical signal generators, noble metal nanoparticles—including gold NPs, silver NPs, etc.—are majorly used in mycotoxins’ (bio)assays due to their unique physicochemical properties. In particular, detection strategies based on changes in the localized surface plasmon resonance (LSPR) signal caused by the aggregation of noble metal NPs have shown suitable sensitivities to detect mycotoxins [23]. In such systems, NPs can be dispersed in colloidal solution via surface anionic repulsion. In the presence of electrolytes containing salt cations or cationic polymers, charges are stabilized, and NPs tend to aggregate. This aggregation alters the LSPR effect, resulting in a red shift of the UV-vis absorption spectrum [24]. Harnessing this property, AuNPs have been extensively tested in the plasmonic sensing of some fungal toxins, owing to their easy synthesis, high extinction coefficients, photostability, and non-toxicity. AuNPs have been considered as ideal signal generating probes because of the visible color change from red to blue through salt-induced nanoparticles assembly, or inversely through their redispersion [23].

**Figure 2:**
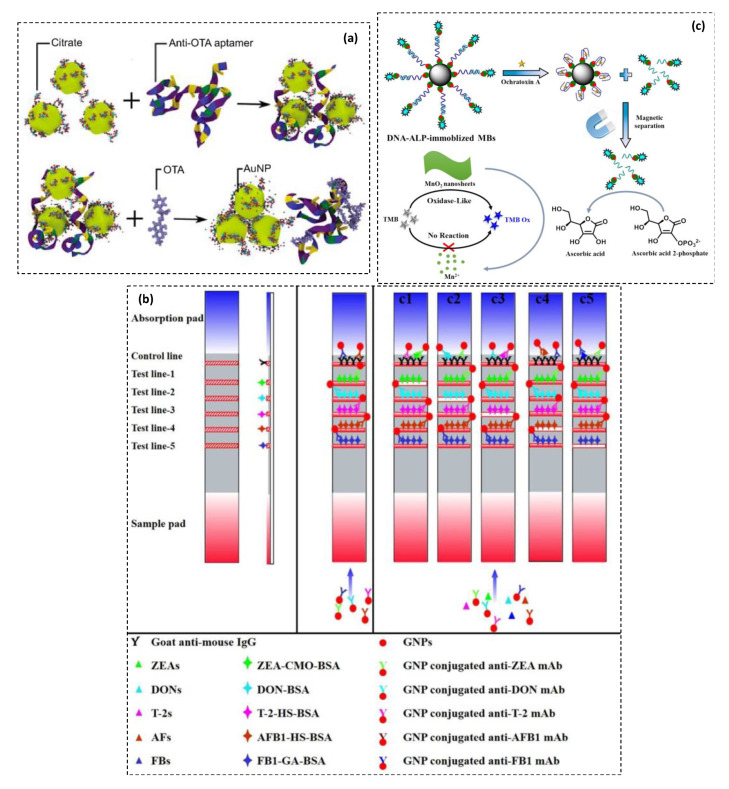
(**a**) Simulation of the molecular interactions involved in the aggregation of citrate-capped AuNPs for the rapid aptasensing of OTA; (**b**) A gold nanoparticle-based semi-quantitative and quantitative LFIA for the simultaneous detection of 20 mycotoxins; (**c**) Mechanism of MnO_2_ nanozyme-based cascade colorimetric aptasensor for OTA detection. Reproduced with permission from [28,33,34].

Specifically, the advances of nucleic acid manipulation and aptamers selection have powerfully accelerated the progress in plasmonic mycotoxins detection [25]. Nucleic acid strands are more convenient than antibodies for unmodified AuNPs aggregation-based assays, with promising results in the semi-quantitative and quantitative real-sample application [26]. For instance, A label-free optical sensor was reported for the selective detection of AFB1 using a DNA-based aptamer along with unmodified spherical colloidal AuNPs (diameter ~ 13 nm). Recognition of AFB1 was achieved based on the salt-induced AuNPs aggregation. High selectivity was observed against the presence of OTA. Low detection-limit of 0.025 ng·mL^−1^ AFB1 was reported with the linear dynamic determination range of 0.025–100 ng·mL^−1^ [27]. More recently, Phanchai et al. [28] have performed in silico studies to investigate the molecular dynamics (MD) of this detection approach using AuNPs aggregation taking as an example anti-OTA aptamer (Figure 2a). This offered new insights into the mechanism of recognition highlighting the effect of the ionic composition of solvent as well as the kinetics behind the interaction between the three molecular partners—i.e., AuNPs, aptamer, and the mycotoxin. The reported MD simulation revealed an insightful analysis of the interaction mechanisms in the AuNP-based aptasensing platforms that can be projected to any other similar pattern.

Another strategy of colorimetric signal generating relies on nanomaterial-based labels like common in lateral flow immunochromatographic assays (LFIAs). A number of NMs was described as antibodies’ nano-labels for the visual rapid detection of mycotoxins [29], such as AuNPs [30], graphene oxide (GO) [31], Prussian blue nanoparticles (PBNPs) [32], etc. In such devices, the color of test lines is usually drawn by the labeled antibodies involved in specific immunoreactions (cf. Section 3.3). Typical mycotoxins’ LFIAs use AuNPs as convenient nano-labels. Interestingly, Kong et al. [33] described a semi-quantitative and quantitative AuNPs-based LFIA for the simultaneous detection of 20 types of mycotoxins from five classes—including zearalenones, deoxynivalenols, T-2 toxins, aflatoxins, and fumonisins—in cereal food samples (Figure 2b). The whole detection process took 20 min in total and was used for the reliable detection of mycotoxins in cereal samples. The LOD of three mycotoxins (AFB1, ZEN, and OTA) were far below the European maximum residue limits.

Nanozymes are unique nanomaterials that have been proven to show catalytic activities in a similar way to biological enzymes with greater stability. This particular feature enables the enhancement of enzymatic response or the development of enzyme-free colorimetric methods. Accordingly, some nanozymes owing peroxidase-like and oxidase-like activities have been used as colorimetric probes for mycotoxins analysis [35]. They are widely used in different formats to afford rapid colorimetric observation, sensitive response, and cost-effective analysis.

For example, Tian et al. [34] developed a sensitive OTA aptasensor harnessing the oxidase-mimicking activity of MnO_2_ nanosheets to catalyze the TMB oxidation (Figure 2c). In this assay, ascorbic acid generated under ALP action reduces MnO_2_ nanosheets to Mn^2+^ ions, and thereby inhibits the catalytic activity of MnO_2_ in the presence of TMB. With the increasing amount of OTA, a highly sensitive color change from blue to colorless was obtained. This sensing method enabled competitive LOD (0.07 nM) compared to conventional single enzymatic colorimetric schemes. While in the colorimetric sensing system based on peroxidase-like nanomaterials, the detection of targets is performed through measuring the absorption variation of the TMB-H_2_O_2_ reaction. According to this scheme, analysis of OTA has been demonstrated using a hybrid recognition matrix composed of Fe_3_O_4_ doped with AuNPs, amino-modified capture DNA and anti-OTA aptamer deposited on glass beads (GB-aptamer/cDNA-Au@Fe_3_O_4_) [36]. The peroxidase-like activity of Au@Fe_3_O_4_ NPs was effectively enhanced due to the synergistic effect between the AuNPs and Fe_3_O_4_ NPs. Low detection limit of 30 pg mL^−1^ OTA was achieved with a linear current response range of 0.05–200 ng·mL^−1^. Selectivity has been proven in the presence of OTB, FB1, and AFB1. Sensor performance for the determination of OTA from real samples has been demonstrated with peanut and corn samples.

It is worth noting that other detection strategies can implicate NMs as signal mediators in combination with other main colorimetric probes for sensitivity enhancement. As an example, MnO_2_ nanosheets can be used in new colorimetric methods based on AuNPs aggregation schemes since MnO_2_ nanoflakes can produce abundance metal ions Mn^2+^ after decomposition [37], or combined with enzymes to react with catalysis products [34].

Representative examples of NMs-based assays from the recent literature are summarized in Table 2.

## 3. Colorimetric Strategies for Mycotoxins Detection

Colorimetric assays in mycotoxins detection have attracted much attention due to their simple sensing mode where colorimetric signal can be detected by the naked eye and without the need to complicated and expensive instruments. They are developed in two modes including solution-based and flat substrate-based assays. Solution-based assays involve free colloidal reagents in the same homogenous phase of targets. Such mycotoxin detection strategies can be performed using organic dyes, colored enzymatic products, or nanomaterial probes. On the other hand, three kinds of colorimetric flat substrate-based assays are more common in mycotoxin detection. They include enzyme-linked immunosorbent assay (ELISA), lateral flow assays (LFAs), and microfluidic-based assays.

### 3.1. Solution-Based Assays

In-solution assays rely on the colloidal interaction between different biomolecules without their immobilization on a substrate. The colorimetric signal is usually generated after cascade additions of reagents in a total volume of some hundreds of microliters where the colorimetric probe, the bioreceptor, and the target can meet. Numerous reports of homogenous solution-based assays for mycotoxins detection have been developed owing to their rapid operation and facile design. Most of these patterns are based on target-induced/disabled nanoparticles aggregation, enzymes or enzyme-like catalytic activities, or chemical dyes in label-based or label-free formats.

As described earlier, noble nanoparticles are characterized by an intrinsic size- and distance-dependent optical signal. Particularly, AuNPs showed a great success in the design of solution-based colorimetric assays using different aggregation approaches.

As an example, a simple colorimetric assay has been described by Chotchuang et al. for the detection of fumonisin B1 using dispersed cysteamine-functionalized gold nanoparticles (Cys-AuNPs) [42]. The target mycotoxin was first hydrolyzed (HFB1) to then induce NPs aggregation via hydrogen bonding. At an optimal pH of 9, color change from wine-red to blue-gray and absorption spectra from 520 nm to 645 nm can be either observed visually or measured by a UV-vis spectrophotometer. This 3 min sensing approach achieved satisfactory results between 2–8 μg kg^−1^ FB1 concentration range and a detection limit of 0.90 μg kg^−1^. Although this method was successfully applied to corn samples, its specificity could be decreased in the presence of interfering molecules that are able to aggregate AuNPs in the absence of target. Therefore, the use of specific bioreceptors such as aptamers is more common in AuNPs-based detection of mycotoxins.

Nucleic acid strands are known to protect AuNPs against salt-induced aggregation because of strong van der Waals interactions between DNA bases and gold [48]. This electrostatic affinity induces aptamer’s adsorption which stabilizes the dispersed nanoparticles. Upon mycotoxin recognition, aptamers desorb from the surface of AuNPs to preferentially complex with the target. Subsequently, stable gold aggregates are formed under the action of electrolytes or cationic polymers leading to the solution color changing. According to this aptasensing strategy, label-free AuNPs-based aptasensors were frequently reported for the rapid detection of mycotoxins [49], including ochratoxin A [38,40,50], aflatoxin B1 [27,51], and zearalenone [52].

**Figure 3:**
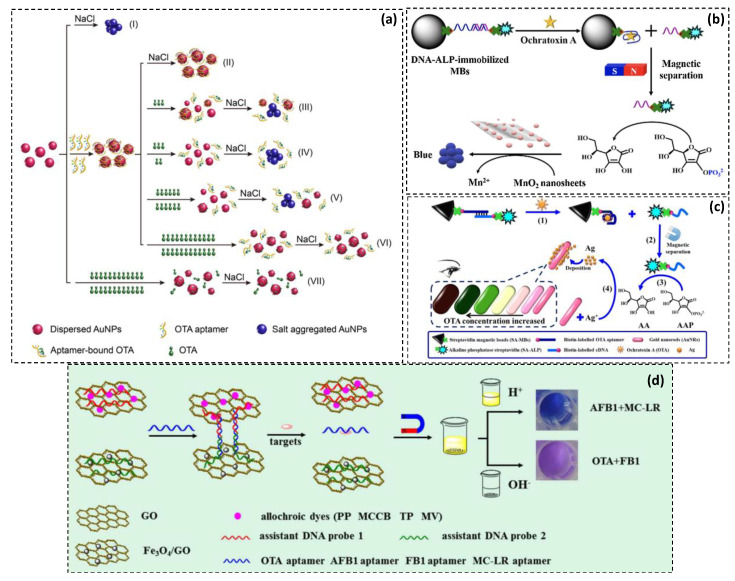
(**a**) Gold nanoparticle-aptamer-based LSPR sensing of ochratoxin a at a widened detection range by double calibration curve method; (**b**) Colorimetric aptasensor for the ochratoxin A (OTA) assay based on the structure-switching of OTA aptamer coupling with alkaline phosphatase (ALP)-MnO_2_ cascade catalytic reaction; (**c**) Multicolor colorimetric detection of OTA via structure-switching aptamer and enzyme-induced metallization of gold nanorods; (**d**) pH-Resolved simultaneous detection of four targets based on magnetic separation of two GO platforms with allochroic dyes. Reproduced with permission from [37,40,53,54].

Interestingly, Liu and collaborators have found that aromatic targets sch as ochratoxin A can also adsorb on the surface of AuNPs after aptamer folding and further inhibit salt-induced aggregation [40]. This limitation renders the assay unreliable at high target concentrations. To expand the detection range, they described a double calibration curve method in which both aggregation mechanisms are combined using two experimental conditions (Figure 3a). Using this system, they claimed that the OTA concentration range could be widened from 10^−10.5^–10^−8^ to 10^−10.5^–10^−6.5^ g·mL^−1^.

Although the achieved limits of detection are demonstrated to be in compliance with regulatory levels, such colorimetric assays present relatively high LOD values compared to other optical or electrochemical approaches. The lack of sensitivity was explained by the number of NPs required to generate a significant color change. Aiming to overcome this constraint, Xiao et al. [39] described a colorimetric aptasensor based on the disassembly of aggregates of oriented AuNP dimers by target molecules. This AuNPs dimer-based sensor has shown better stability, sensitivity (LOD = 0.02 µg·L^−1^) and OTA detection dynamic range (0.08–100.8 µg·L^−1^). Furthermore, it was noted that the disassembly of AuNPs dimers was faster than that of large aggregates reducing thus the analysis time [41].

In another option, the colorimetric signal of substrate-free assays can be amplified by catalytic reactions using either enzymes or nanozymes. Harnessing the inherent peroxidase-like activity of AuNPs, Sun et al. [41] developed a rapid apta-assay specific to zearalenone. In this assay, ZEN aptamer inhibits the catalytic activity of AuNPs in the presence of H_2_O_2_ and TMB. The solution remains red until the target binding to the aptamer, which restores the peroxidase-mimicking of nanozymes to oxidize the colorless TMB into blue oxTMB. Quantitative analysis was reported in the ZEN concentration range of 10–250 ng·mL^−1^, and the limit of detection is 10 ng·mL^−1^. The assay was applied to test ZEN in corn and corn oil samples, but high sensitivity was still challenging.

As an alternative, a combination of enzymatic action and gold nanoparticles aggregation was suggested by He et al. [37]. This colorimetric method was developed to detect OTA indirectly via the activity of alkaline phosphatase (ALP) (Figure 3b). Briefly, aptamer-modified magnetic beads (MBs) were conjugated to DNA-linked ALP by hybridization. After OTA recognition, magnetic separation allowed to collect the released quantity of enzyme. The ALP can then hydrolyze ascorbic acid 2-phosphate (AAP) to ascorbic acid (AA), which mediates the reduction of MnO_2_ nanosheets to Mn^2+^. These metal cations allow thereby the aggregation of AuNPs and lead to vivid color changes in the sensing system [37]. The dynamic range extends from 6.25 to 750 nM and an improved LOD of 2 ng·mL^−1^ was recorded. This colorimetric method was applied to grape juice and red wine matrix with satisfactory recoveries.

A comparable approach was also described by the same group while replacing MnO_2_ nanosheets by gold nanorods (AuNRs) and silver ions [53]. After magnetic separation, generated AA acted as reducing agents that transform Ag^+^ to metal silver forming an Ag shell on the surface of AuNRs (Figure 3c). This caused a blue-shift of the longitudinal AuNRs’ LSPR and a rainbow-like multicolor change.

Multicolor detection of OTA was also reported by AuNRs etching (diameter ~ 14 nm) mediated by G-quadruplex (AG4-OTA)-hemin DNAzyme and exonuclease I [55]. The product of peroxidase-like activity in acidic solution TMB^2+^ can etch the AuNRs by oxidizing Au(0) into Au(I).Variation of the optical characteristics of AuNRs arising from the change in interparticle distance and the number of hydrogen bonds has been reported as the key sensing strategy. A linear response range of 10–200 nM OTA was found with a LOD of 30 nM by visual observation and a lower LOD of 10 nM by spectrophotometry. The selectivity towards OTA was tested with the interfering mycotoxins AFB1, ZEN, and OTB. The method was successfully applied to the determination of OTA in spiked beer samples.

Besides enzymes and nanomaterials, commercially available organic dyes have also been used to conceive solution-based colorimetric methods. Interestingly, Wang’s research group developed some multiplexed assays for the real-time detection of different mycotoxins based on allochroic dyes [45,54]. For instance, Hao et al. proposed a pH-resolved colorimetric aptasensing method for the simultaneous detection of four targets, including three mycotoxins, ochratoxin A, aflatoxins B1, fumonisin B1, and a marine toxin, microcystin-LR [54]. This assay involves four allochroic dyes—namely, phenolphthalein (PP), malachite green carbinol base (MGCB), thymolphthalein (TP), and methyl violet (MV)—as multiple signal indicators with colors of different wavelengths. Two DNA-GO platforms were prepared; the first was modified with Fe_3_O_4_ for magnetic separation while the second adsorbed the hydrophobic dyes (Figure 3d). Both platforms were linked by partial hybridization to a target-specific aptamer. Upon target recognition, aptamer structure switching disabled hybridization and dissociated GO platforms. The subsequent magnetic separation followed by centrifugation allowed the spectroscopic analysis of supernatant in acidic solution and precipitate in alkaline solution. The absorption of supernatant solutions was directly proportional to AFB1 and MC-LR concentrations because of the MGCB and MV release at pH 3. Whereas the absorption of precipitates containing PP and TP adsorbed dyes was inversely proportional to OTA and FB1 mycotoxins in an alkaline pH of 12. This approach enabled the simultaneous detection of OTA and AFB1 in peanut samples with satisfactory recoveries (97.8–104.3%).

More recently, a derived nanocomposite-based strategy was described by Zhu et al. [45] employing TP dye signaling in acidic conditions for AFB1 detection and AuNPs as nanozymes to detect OTA via TMB catalysis in the alkaline precipitate. Competitive limits of detection as low as 1.5 ng·mL^−1^ and 0.15 ng·mL^−1^ were thus obtained for AFB1 and OTA, respectively.

### 3.2. Enzyme-Linked Immunosorbent Assay (ELISA)

According to the literatures, ELISA is the most popular and most frequently used technique for mycotoxin analysis especially aflatoxins [56]. Among different types of ELISA, the direct competitive ELISA is commonly used in mycotoxin detection. In recent years, only a few studies have been focused on mycotoxin detection using ELISA method. However, there are a large number of commercial ELISA kits produced by different companies worldwide.

**Figure 4:**
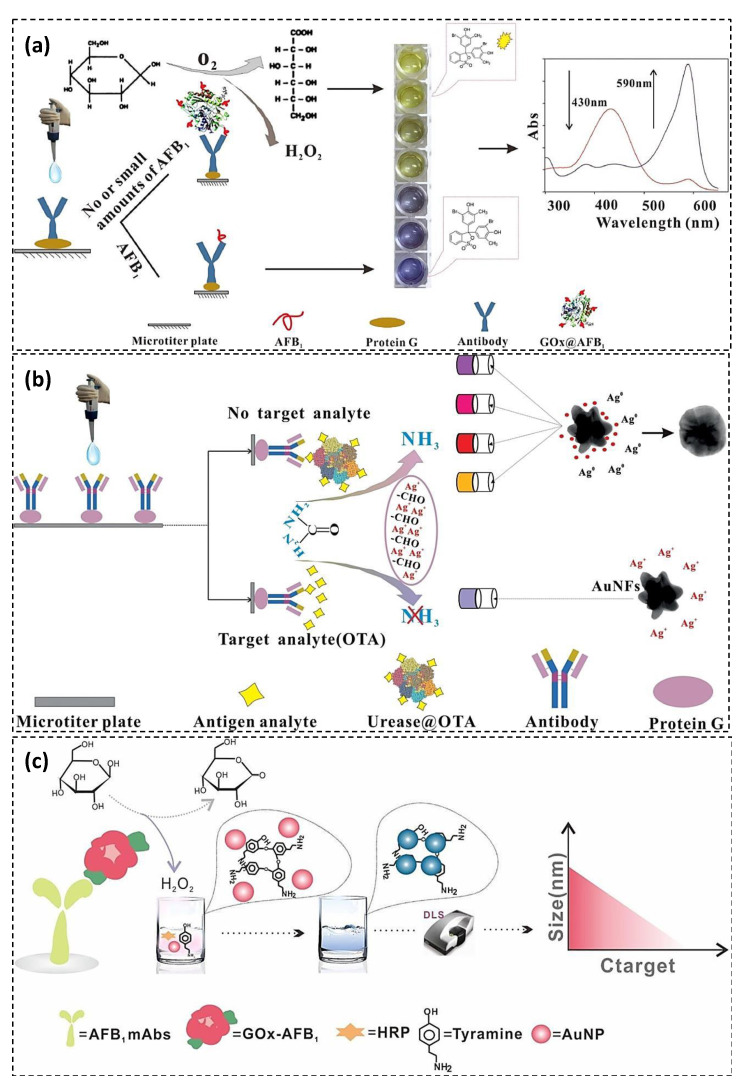
(**a**) Colorimetric GOx-based ELISA using acid-base indicator bromocresol purple (BCP) for AFB1 detection; (**b**) Plasmonic ELISA based on the urease-induced metallization of gold nanoflowers for OTA detection; (**c**) DLS-ELISA method associated with H_2_O_2_-mediated tyramine signal amplification system for AFB1 detection. Reproduced from [58,60,61], respectively, with permission.

Traditional ELISA uses antibody as recognition element and HRP-catalyzed TMB to generate color as a signal reporter. Although ELISA has been recognized as an excellent and accurate method for mycotoxin analysis, but the procedure is somewhat time-consuming (incubation time of approximately 1–2 h), uneconomical, unsuitable for field testing due to the need for specialist plate readers, and unreliable due to the similarity of the structure of mycotoxins, which causes false positive results [57]. Therefore, many efforts have been made to improve the shortcomings mentioned. One attempt is to improve the colorimetric signal. Conventional colorimetric signal using HRP and TMB is not suitable for naked-eye detection in deprived areas with limited resources because a plate reader is required to distinguish the tonality of analytes with similar concentrations. Recently, colorimetric ELISA has gained considerable attention due to its simple readout without specialist devices. Acid–base indicators are ideal signal reporters for naked-eye distinction because most of them provide a significant contrasting color at their titration end points under a narrow pH range. Several enzymes including alkaline phosphatase, urease and penicillinase have been used in ELISA to change the pH through catalyzing the related specific substrate to produce hydrogen or hydroxide ions [58]. In this regard, Xinog et al. developed a direct competitive colorimetric ELISA using glucose oxidase (GOx) as an alternative to HRP for glucose oxidation into gluconic acid and H_2_O_2_ (Figure 4a) [58]. The pH indicator bromocresol purple (BCP), which was highly sensitive to pH variation, was applied as signal output. BCP indicator showed a vivid color change from yellow to grayish purple in the presence of 100 pg·mL^−1^ AFB1. Therefore, the cutoff limit was determined to be 100 pg·mL^−1^ by the naked eye. The developed GOx-based colorimetric ELISA exhibited high sensitivity and excellent selectivity with IC50 value at 66.27 pg·mL^−1^, which was approximately 10-fold lower than that of traditional HRP-based ELISA. However, long incubation time and multi-step washing were still the major limitations of the ELISA method. The proposed assay was applied for AFB1 determination in corn samples with acceptable accuracy and precision.

Among colorimetric ELISA methods, plasmonic ELISA is another attempt with simple readout format suitable for on-site detection. Gold nanoparticles (AuNPs) are good candidate as colorimetric indicator in plasmonic ELISA due to high molar extinction coefficient and localized surface plasmon resonance (LSPR) characteristics. The LSPR of AuNPs is related to their size, shape, composition, and agglomerate mood [59]. The LSPR variation of AuNPs generates a significant color change that is easily observable by the naked eyes. Based on differences in producing LSPR mechanism, plasmonic ELISA is classified into four types that employ the aggregation, etching, controlled growth kinetics, and AuNPs metallization. Among them, enzyme-induced silver metallization on the AuNPs surface can produce a remarkable LSPR, and provide a multicolor change in the solution [60]. Several enzymes GOx, alkaline phosphatase and β-galactosidase have been used to catalyze their substrates and produce reducing agents such as H_2_O_2_, ascorbic acid, and p-aminophenol which can reduce the silver ions on the AuNPs surface. In this regard, Pei et al. developed a colorimetric plasmonic ELISA for OTA detection based on the urease-induced metallization of gold nanoflowers (AuNFs) [60]. OTA-labeled urease was employed as competing antigen to hydrolyze urea into ammonia. In the presence of ammonia, silver ions were reduced by the formyl group from glucose to produce a silver shell around AuNFs resulted in the solution color change from blue to brownish red (Figure 4b). The plasmonic ELISA exhibited high sensitivity with a cutoff limit of 40 pg·mL^−1^ and LOD at 8.205 pg·mL^−1^ (19-folds lower than those of HRP-based ELISA). The proposed procedure provided a highly selective and sensitive, simple, robust, and high-throughput screening method for the quantitative determination of OTA in food and feed samples. However, it suffered from long incubation time.

In another plasmonic ELISA, aggregation-induced color change of AuNPs, as a main strategy to regulate the plasmonic signal, was employed for the ultrasensitive detection of AFB1 using dynamic light scattering (DLS) signal instead of absorbance (Figure 4c) [61]. In the developed DLS-ELISA, GOx-AFB1 was used as competing antigen because the GOx can effectively convert glucose to H_2_O_2_. Then, the produced H_2_O_2_ converted into hydroxyl radical in the presence of HRP to induce AuNPs aggregation. Indeed, H_2_O_2_-mediated TYR was used as signal amplification system. The DLS-ELISA exhibited a LOD as low as 0.12 pg·mL^−1^ which was about 153- and 385-folds lower than those of conventional plasmonic and colorimetric ELISA, respectively. The ultrahigh sensitivity is attributed to the high sensitivity of light-scattering intensity to particle size changes. The DLS-ELISA was employed for AFB1 detection in corn samples with good reliability and precision.

Mukherjee et al. compared aptamer-based enzyme linked apta-sorbent assay (ELASA) with antibody-based ELISA and its potential to replace antibodies in usual immunoassay formats either as capture probe or detection probe without affecting the sensitivity [62]. The ELASA was based on the principle of target capture by aptamer where, OTA specific aptamer was used for toxin detection. Then, anti-OTA IgG primary antibody and anti-rabbit secondary antibody labeled with alkaline phosphatase (ALP) where added as detection agents. The colorimetric signal was produced under addition of para-nitrophenyl phosphate (p-NPP) as substrate. The LOD was obtained 0.84 pg·mL^−1^. The developed ELASA exhibited a similar sensitivity to the conventional antibody-based ELISA with a LOD of 1.13 pg mL^−1^. However, the OTA aptamer showed about 40% cross-reactivity with aflatoxins. By selecting aptamer with a low percentage of cross-reactivity, ELASA can be a good alternative to the conventional ELISA. The proposed ELASA was used for OTA detection in groundnut and coffee bean.

Another innovation in improving the ELISA characteristics is to replace nanomaterial-based enzyme mimics (nanozymes) as artificial enzymes with natural enzymes. Nanozymes exhibit excellent properties such as easy synthesis, high stability, low cost, and design flexibility. Different kinds of nanomaterials—including noble metal nanoparticles (e.g., AuNP and AgNPs), graphene oxide, magnetic iron oxide, etc.—have been used in sensing methods. Xu et al. proposed a nanozyme-linked immunosorbent assay using metal–organic frameworks (MOFs) for AFB1 detection [63]. MOF with peroxidase-like activity was replaced with HRP for antibody labeling and catalyzing TMB to generate colorimetric signal. The MOF-ELISA system increased the accuracy of detection and inhibited false positive problems in the detection method, indicating that MOFs exhibited better catalytic activity and more stability than HRP. The LOD was obtained 0.009 ng·mL^−1^ which was 20-folds lower than those of HRP-based ELISA. The proposed ELISA was employed for AFB1 detection in peanut milk and soymilk.

Representative examples of recent developed ELISA methods for the detection of mycotoxins are reported in Table 3.

### 3.3. Lateral Flow Assays

Lateral-flow assays (LFAs), also known as immunochromatographic assays (ICAs), are among the most widely used and popular methods in detecting various analytes such as microorganisms, pesticides, heavy metals, diseases biomarkers, and mycotoxins. In recent years, researchers have paid more attention to screening mycotoxins by LFA. LFA is based on the movement of fluid sample across the membrane by capillary force and binding reaction between antibody-antigen or nucleic acid-target analyte [67]. The standard LFA strip is comprised of four parts including a sample pad (the area where the sample is dropped); a conjugate pad (the area where biorecognition element conjugated with label is immobilized); a reaction nitrocellulose membrane (the area containing test line and control line for target binding to antibody or nucleic acid probe); and absorbent pad (as a wick to reserve additional fluid flow) [68,69]. Sandwich mode (for large analytes) and competition mode (for small analytes) are the two most widely used detection formats. Competitive mode is suitable for mycotoxins with low molecular weight and single epitope.

The optimization of the experimental conditions is crucial to develop a LFA with excellent performance and high sensitivity. High sensitivity, low immunoreagent consumption, and ideal color intensity are major parameters for the construction of LFAs. Utilization of an appropriate label is important for a sensitive analysis. Different colored labels such as colored latex beads, AuNPs, magnetic particles (MPs), carbon nanostructure, and enzymes have been used for developing LFA. In addition to sensitivity, label should not change the features of biorecognition element, and it must create stable conjugation with recognition element. AuNPs have been frequently used colorimetric labels in developing LFA strip due to having all the mentioned features [68,69,70]. Di Nardo et al. developed a novel LFA using dual color AuNPs and a single Test line for simultaneous determination of AFB1 and type-B fumonisins (FMBs) [71]. In this assay, red (spherical, mean diameter ≈ 30 nm) and blue (desert rose-like, mean diameter ≈ 75 nm) AuNPs were conjugated to anti-aflatoxin and anti-fumonisins antibodies, respectively. The single test line was formed by spraying the mixture of two antigens including AFB1-BSA and FMB-BSA. According to the competitive format, mycotoxin-free samples provided a purple test line due to the combination of the red and blue AuNPs. Contaminated samples with AFB1 or FMBs resulted in the blue and red color Test line, respectively. The simultaneous presence of both mycotoxins provided the usual disappearance of the Test line. (Semi-) quantitative analysis was obtained using a simple smartphone and RGB colorimetric analysis. The use of a single strip to multiplex analysis provided a simple, rapid, low-cost and reagent-saving assay. The developed strips with LOD at 0.5 and 20 ng·mL^−1^ for AFB1 and FMB1, respectively, were employed to determine these two mycotoxins in wheat and pasta samples.

**Figure 5:**
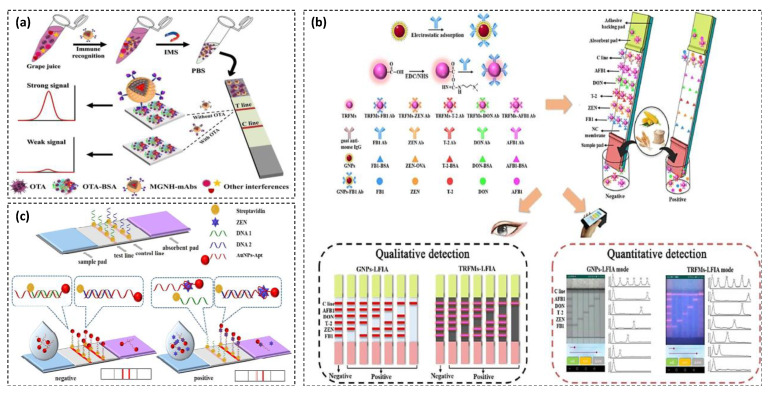
(**a**) Schematic illustration of the detection principle of the MGNH-based LFA strip; (**b**) smartphone-based AuNPs and TRFMs-LFAs for multiplex mycotoxins detection; (**c**) aptamer-based LFA for ZEN detection in the presence and absence of ZEN analyte. Reproduced from [30,72,75], respectively, with permission.

Conventional AuNPs-based LFA suffers from a major challenge in measuring target concentration in complex food matrices with dark color due to its poor resistance to the background matrix and color interference. To address this issue, Hao et al. developed a novel LFA using bifunctional magneto-gold nanohybrid (MGNH) label as a hetero-structured nanomaterial for the simultaneous magnetic separation and colorimetric detection of OTA in grape juice [72]. In this assay, MGNH-labeled monoclonal antibodies (mAb) were used for the MGNH-mAb-OTA complex formation and subsequently rapid separation of the complex from sample using an external magnetic field. Then, MGNH-mAb-OTA complex was resuspended in buffer and applied on LFA strip for colorimetric detection (Figure 5a). Grape juice with purple color and high concentrations of sugar, pigment, and tannins was used as complex matrix to evaluate the designed method. The novel LFA was highly sensitive with LOD at 0.094 ng·mL^−1^. The assay showed high accuracy, reproducibility, practicability, and short detection time (10 min of magnetic separation and 5 min of immunoreaction).

Most of the multiplex LFAs for mycotoxins analysis have been designed for detection of only two or three kinds of mycotoxins [73], while sometimes more than this occurs in some foods such as cereals. On the other hand, quantitative analysis is a main issue in LFA technology which is often carried out by desktop readers or handheld readers. These devices are slightly inferior in terms of popularity, portability, and timely data sharing compared to smartphone-based analysis [74]. Therefore, these existing limitations must be overcome to receive a practical LFA for multiplex and on-site detection. For this purpose, Liu et al. developed two kinds of multiplexed LFA strips using AuNPs and time-resolved fluorescence microspheres (TRFMs) as label for the detection of AFB1, zearalenone (ZEN), deoxynivalenol (DON), T-2 toxin (T-2), and fumonisin B1 (FMB1) in cereals (Figure 5b) [30]. Five test lines were sprayed on a single test strip for each mode of detection. Quantitative results were obtained using a smartphone dual detection mode device. The visual LODs of AuNPs-LFA were 10, 2.5, 1, 10, and 0.5 ng·mL^−1^ for AFB1, ZEN, DON, T-2 and FMB1, respectively. In the TRFMs-LFA format, LODs were 2.5, 0.5, 0.5, 2.5, and 0.5 ng·mL^−1^, respectively for the mentioned mycotoxins. Quantitative LODs (qLODs) for these mycotoxins were obtained 0.59, 0.24, 0.32, 0.90, and 0.27 ng·mL^−1^ (in AuNPs-LFA), and 0.42, 0.10, 0.05, 0.75, and 0.04 ng·mL^−1^ (in TRFMs-LFA). TRFMs-LFA was more sensitive than AuNPs-LFA due to large surface area and stokes shift of TRFMs. On the other hand, AuNPs was low-cost, more popular, stable and easy to synthesize. The assay was reliable, quantitative and highly sensitive for on-site detection of multiple mycotoxins. However, a main problem of a multiplex LFA is the cross reactivity between Ag-Ab pairs, so that the developed LFA was able to detect 20 mycotoxins from five classes.

In addition to AuNPs, colloidal carbon can be used as a colored label in LFAs. It is comparatively inexpensive and can be synthesized in a large scale. Furthermore, it shows high chemical stability and recognizable color to develop LFA with high sensitivity. Many colloidal carbon-based-LFA have been developed for detection of different analytes. For example, Yu et al. proposed a LFA using Graphene oxide (GO) and carboxylated GO as labels for AFB1 detection [31]. GO can be easily conjugated with biomolecules without any additional activation due to having a large variety of oxygen-containing chemical groups. Moreover, it shows excellent hydrophilicity and high stability at room temperature. In this study, mA against AFB1 was conjugated with GO. The vLOD and cut-off values for AFB1 were 0.3 and 1 ng·mL^−1^, respectively. It was exhibited that GO and carboxylated GO can be used as viable black labels to develop a low-cost LFA compared to the AuNPs labels. The method was successfully applied for AFB1 detection in peanut oil, maize, and rice.

In recent years, many efforts have been made to replace aptamer with antibody in LFA technology due to potential advantages of aptamers (as mentioned earlier). However, aptamer-based assays mostly require laboratory infrastructure, which limits their application. Incorporating the advantages of aptamers and LFA technology is a basic step to complete the user-friendliness of aptamers. In this context, Wu et al. developed a competitive aptamer-based LFA for rapid and sensitive detection of ZEN [75]. The assay was based on competition between the DNA 1 on the test line and ZEN in the for binding to AuNPs-labeled aptamer. In the absence of ZEN, AuNPs-labeled aptamer hybridized with DNA 1 on the test line and DNA 2 on the control line, resulting in two colored lines on the strip while, in the presence of high concentration of ZEN, the test line was colorless (Figure 5c). The proposed aptamer-based LFA with high specificity and sensitivity (vLOD and qLOD of 20 and 5 ng·mL^−1^, respectively) and short detection time (5 min) was applied for ZEN detection in spiked corn samples.

Representative examples of recent developed LFA test strips for the detection of mycotoxins are reported in Table 4.

### 3.4. Microfluidics-Based Assays

In addition to LFA, microfluidic technology has attracted much attention in recent years to detect a variety of analytes. According to the definition provided by Whitesides from Harvard University, “microfluidic is the science and technology of systems that process or manipulate small amounts (10^−9^ to 10^−18^ L) of fluids, using channels with dimensions of tens to hundreds of micrometers”. This technique shows a great potential to control the concentrations of molecules in space and time [82]. High surface-to-volume ratios, small consumption of reagents, prevalence of viscous and capillary forces and laminar flows are the major features of microfluidic-based systems [83]. Based on such properties, microfluidic can be integrated with biosensor technology in order to develop analytical devices with high sensitivity, reproducibility, portability, low-cost, short detection time, and high throughput [84]. Early microfluidic systems were fabricated of silicon and glass. Because of high cost of silicon and fragility of glass, polymer-based devices were then offered in the late 1990s which were cheaper than glass and silicon and provided an extensive range of chemical materials expanded from polydimethylsiloxane (PDMS) to thermoplastics [85,86].

**Figure 6:**
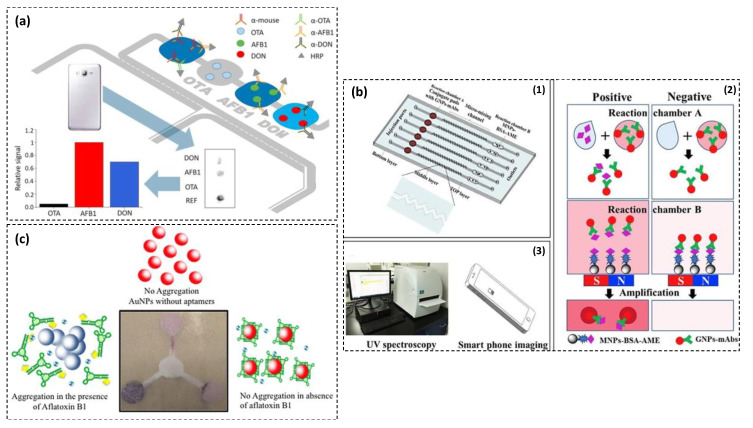
(**a**) Schematic representation of the microfluidic immunoassay with smartphone data acquisition for multiplexed mycotoxin detection; (**b**) microfluidic immunoassay for AME detection: (**1**) The 3D structural diagram of the fabricated microfluidic chip, (**2**) The schematic of the AME detection using the amplified microfluidic immunoassay, and (**3**) The colorimetric detection by UV spectroscopy and smartphone imaging APP; (**c**) Development of a µPAD using aptamer and for AFB1 detection based on salt-induced aggregation of AuNPs in the presence of analyte. Reproduced from [83,87,88], respectively, with permission.

Recently, microfluidic-based assays have attracted a large amount of interest in the detection of mycotoxins. The incorporation of microfluidic system and immunoassay is considered as one of the most popular platforms for detecting mycotoxins with high sensitivity and short detection time. For example, Machado et al. developed a PDMS-based microfluidic immunoassay with four chambers for simultaneous detection of OTA, AFB1, and DON [83]. The competitive immunoassay was developed by the immobilization of BSA-mycotoxin conjugates onto separate chambers (Figure 6a). The first inlet was considered for sample loading. In the presence of the given mycotoxin, the free toxin competed with the toxin-BSA immobilized on the PDMS surface for the specific binding to IgG-HRP conjugate. Therefore, a high concentration of a target free toxin resulted in a low density of IgG-HRP captured by the immobilized BSA-toxin. After addition of TMB, a colorimetric signal was observed which was inversely proportional to the mycotoxin concentration. Smartphone was used to obtain semi-quantitative results. The proposed assay exhibited LODs at <40, 0.1–0.2 and <10 ng·mL^−1^ for OTA, AFB1, and ZEN, respectively. Furthermore, the immunoassay was applied for the simultaneous detection of these three mycotoxins in corn samples after a simple sample preparation method. The multiplexed analysis with a relatively low cost and simple operation can be performed in less than 10 min. However, these methods can be simplified by reducing the number of user-intervention steps such as pipetting.

In another microfluidic device, AuNPs were used as colored labels for indicating various concentrations of alternariol monomethyl ether (AME), one of the most frequently occurred Alternaria mycotoxins [87]. Microfluidic chip was fabricated using Norland Optical Adhesive 81 and glass substrate (Figure 6b). AuNPs a conjugated with AME specific mAb and magnetic nanoparticles (MNPs)-BSA-AME conjugates were used as capture probe and competitive antigen, respectively. In the presence of AME, it firstly bound to the AuNPs-mAbs in conjugate pad and micro-mixing channel. Therefore, large numbers of free AuNPs-mAbs-AME conjugates were kept in supernatant after magnetic separation. Then, the supernatant was transferred into immunogold amplification solution containing ascorbic acids as reducing agent and hexadecyltrimethylammonium bromide as a surfactant to stabilize the amplified AuNPs-mAbs. In this solution, the free AuNPs-mAbs-AME conjugates were used as gold seeds for the signal amplification. UV spectroscopy and smartphone imaging APP were used for monitoring of the AuNPs color change. The assay was able to analyze six samples in parallel within 15 min. The fabricated microfluidic immunoassay exhibits LODs at 12.5 pg·mL^−1^ and 200 pg·mL^−1^, by UV spectroscopy and smartphone imaging, respectively. It was successfully applied for AME detection in spiked fruit samples. The device can be used for sensitive, rapid, low-cost, and on-site detection of mycotoxins.

Paper is an ideal substrate to construct microfluidic devices. It is a good alternative to glass and polymer. Paper-based microfluidic systems were introduced by Whitesides group in 2007 as lab-on-chip (LOC) devices. The paper-based microfluidic devices are cheaper, easier to fabricate, easier to use, easier to dispose, compatible to chemicals/biochemicals used in bio-medical applications and environmentally friendly. Paper segmentation to hydrophobic and hydrophilic regions by hydrophobic materials can provide hydrophilic channels for fluid flow via capillary action and without the need for a pump. However, despite all these advantages, paper-based microfluidic devices are only suitable for semi-quantitative rather than quantitative analysis [85,86,89].

Although there are a number of well-developed systems for immunoassay in microfluidic (lab-on-chip) format, the use of aptamers in similar devices is on very beginning. Kasoju et al. developed a microfluidic paper-based analytical device (µPAD) for AFB1 detection using aptamer as recognition element [88]. The hydrophobic barriers were developed on the Whatman filter paper using photolithography. Two control zones (negative and positive) and one analyte zone were designed on the paper. Detection was performed based on salt-induced aggregation of AuNPs in the presence of analyte. The aptamer-AuNPs conjugate was adsorbed onto the paper through physical adsorption and AFB1 was allowed to flow over the µPAD. In the presence of AFB1, the aptamer combined with AFB1 and bare AuNPs was aggregated in the presence of NaCl (Figure 6c). The developed assay showed a LOD of 10 nM in spiked samples. The developed µPAD was suitable for rapid (detection time < 1 min), simple, label-free, and on-site detection of mycotoxins.

Representative examples of recent developed microfluidic-based assays for the detection of mycotoxins are reported in Table 5.

## 4. Recent Advances toward Practical Applications

Currently there are several reference methods for mycotoxin analysis such as HPLC and LC-MS/MS. However, their usage is dependent on a variety of factors including expensive laboratory equipment, academic laboratories with skilled personnel, and time-consuming analysis [10]. In these cases, biosensors and rapid detection kits can overcome such limitations. The primary goal in developing a biosensor or bioassay is making an analytical device that can rapidly and accurately quantify target analytes in the field at a low cost. A biosensor/bioassay with such features can have the potential to be practical and commercial. As described above, the colorimetry sensing technology is springing up showing excellent sensitivity as a powerful tool for mycotoxins detection. However, sample pretreatment is fundamental for the extraction of mycotoxins from complex food and feed matrices. This preliminary step is necessary due to many interfering compounds—such as proteins, lipids, sugars, and salts in complex food/feed samples—leading to matrix effects, signal interference, instruments contamination, and even false-positive results [91].

### 4.1. Mycotoxin Extraction Methods

Food and feed sample pretreatment mainly requires the selective isolation and enrichment of target analytes from the complex matrix. After the size reduction of solid samples, extraction of mycotoxins can be performed. Most of the mycotoxins are soluble in organic solvents—including methanol, acetone, acetonitrile, ethyl acetate, and dichloromethane—but are hardly soluble in water except fumonisins and patulin [92]. Solvent extraction is the most common method for the extraction and purification of mycotoxins in colorimetric assays. In a representative extraction procedure, liquid samples such as milk, juice, and wine are directly subjected to liquid-liquid extraction for the initial isolation of mycotoxins. However, solid-liquid extraction is used for the extraction of mycotoxins from solid samples such as grains and cereals. Generally, a mixture of organic solvent and acidic buffer or water is extensively used to extract mycotoxins. In this mixture, the water improves the organic solvent’s penetration in the food/feed matrix, while the acidic solvent can decompose the strong bonds between the analyte and other food components (e.g., protein and sugars), resulting in increasing the extraction efficiency [93].

Because of the destructive effect of organic solvents on enzyme or antibody bioreceptors (e.g., denaturing properties), many efforts have been made to minimize these effects or replace new extraction methods. In the first case, diluting the sample after solvent extraction can reduce the solvent’s damaging effect. In the latter case, supercritical CO_2_ extraction and microwave-assisted extraction can be promising methods for replacement with conventional solvent extraction.

After mycotoxin extraction, filtration and centrifugation are used to remove any suspended particles. In most cases, no further purification steps are required, and the sample is used for detection. However, in biosensors/bioassays with high detection limits, clean-up procedures such as solid-phase extraction (SPE), dispersive solid-phase extraction (DSPE), solid-phase micro-extraction (SPME), and immunoaffinity column (IAC) can be performed in order to improve the detection sensitivity and specificity [9]. These advanced extraction methods were recently reviewed by Agriopoulou et al. [94].

### 4.2. Inventory of Commercially Available Kits for Mycotoxins Detection

Commercial test kits for mycotoxin detection are utilized as an appropriate alternative for more user-friendly, inexpensive, robust, and rapid analysis. There are a large number of commercial detection kits available for mycotoxin analysis in the current market, as summarized in the Table 6. They commonly include ELISA kits, membrane-based immunoassays such as lateral flow immunoassays (LFIAs), fluorescence polarization immunoassays (FPIAs), and immunoaffinity column coupled with fluorometric assay. The majority of these test kits are based on an immunoassay format which relies on the specific interaction of antigen and antibody. Moreover, colorimetric detection kits are most preferred because of the ability to see results with the naked eye. Among them, LFIAs with acceptable sensitivity, good accuracy, portability, short detection time, ease of use, and no need for specialized personnel have become strong competitors on the market for mycotoxin analysis. Several companies worldwide produce LFIA test strip to analyze different kinds of mycotoxins. Charm Sciences Inc., Pribolab, EnviroLogix, Romer labs, Vicam, CUSABIO, etc. are among LFIA strips producer for aflatoxins, DON, ZEN, T2, and OTA. ROSA (rapid one step assay) lateral flow strips developed by Charm Sciences Inc. are the leading mycotoxin test worldwide.

Commercial LFIAs test strips usually use AuNPs as colored label. The method can provide qualitative and/or semi-quantitative results within minutes (e.g., Afla-V and AflaCheck strip tests by Vicam, 5 and 3 min, respectively). For semi-quantitative analysis, portable readers have been developed for on-site detection. For example, PerkinElmer’s QuickSTAR Horizon strip reader provides quantitative results for mycotoxins including AFB1, AFB2, AFG1, and AFG2; at detection levels of 2 to 300 ppb within 6 min. Charm EZ-M Reader is another type of portable strip reader which can show results within 3–5 min. The color-coded strips allow the EZ-M reader to automatically recognize which mycotoxin group you are testing and will adjust the reader temperature and time accordingly. Some companies such as R-Biopharm provided a mobile app on a smartphone to analyze color signal instead of a reader, specifically for aflatoxins, T2/HT2, ZEN, and FMN.

Commercially available test kits have been developed for determination of individual mycotoxins or for multiple mycotoxins in one group (e.g., aflatoxins B1, B2, G1, and G2). Since we are usually faced with contamination of food and feed to more than one mycotoxin, the current trend in LFIA technology is to develop strips with multiple test lines for the simultaneous detection of multiple mycotoxins.

After LFIA test strips, ELISA-based kits have allocated a major portion of market amongst other mycotoxin detection methods. Many companies—such as Sigma, Elabscience, Eurofins, Romer Labs, ELISA Technologies, Cusabio, Astori Tecnica, etc.—offer ELISA kits to detect the most common types of mycotoxins. Some of these kits can detect several types of mycotoxins in one group (e.g., aflatoxins B1, B2, G1, and G2).

The HRP-TMB system is the most common method for colorimetric signal generation in ELISA-based kits. Commercial ELISA kits are sensitive, selective, high throughput, with minimum sample preparation steps. Moreover, the detection time in commercial kits has been shortened so that, most of them are able to detect target mycotoxin within 1–2 h. Romer labs has produced ELISA kits for aflatoxins, OTA, DON, T2, ZEN, and FMN with incubation periods of 15 min. In some kits, cross-reactivity of antibodies leads to overestimation of results while matrix effect plays a key role in providing false-positive results. To avoid such effects, most kits define the limited matrices to which the ELISA kit can be used [115]. However, this feature can be considered a limitation for such ELISA kits. On the other hand, some companies such as Eurofins have developed sensitive ELISA method for a wide range of matrices.

As with the LFIA kits, the current trend in ELISA technique is to develop commercial multiplex assays. Multiplex ELISA can be developed by immobilizing different toxins in different wells of a single microplate in competitive format.

### 4.3. Interesting Examples from Literature with Great Potential for Industrial Applications

As it can be observed from literatures, there are many new studies on mycotoxin detection with the potential for industrial application. However, several parameters should be considered before a bioassay or biosensor can reach the commercialization stage. These parameters are briefly described in the following.

LOD and sensitivity: Sensitivity is a main factor in mycotoxin detection due to the presence of mycotoxins in small amounts in food and their toxic hazards to the consumer in very low concentrations. Regarding this issue, most of developed biosensors/bioassays show high sensitivity which makes them suitable for point of care testing (POCT) application. For quantitative assays, the LOD is defined as the mean value of the blank (matrix blank) reading in analyte concentration, plus three times the standard deviations [115]. In the case of sensitivity, false negative and false positive results and sample matrix which can affect the assay sensitivity should be considered.

Specificity and cross reactivity: Since antibodies are the most used recognition element in the development of mycotoxin bioassays, the use of monoclonal antibodies with high specificity and low cross-reactivity is of great importance in the development of commercial kits. Cross-reactivity of antibodies can lead to overestimation of results and inaccurate overall risk assessment for consumers. Generally, individual mycotoxin assays show higher specificity compared to multiplex assays. Because detection of multiple mycotoxins with a single test an important feature in developing commercial devices, the use of antibody with acceptable cross-reactivities to detect groups of related mycotoxins can be affordable.

Accuracy and precision: accuracy and precision are important parameters for practical application of a developed assay. Accuracy is proximity of the measurements to a specific value obtained by a reliable method. A method for expressing the accuracy of the developed analytical approach is by establishing a correlation between results of the developed and the reference methods. In the case of mycotoxin detection, HPLC or LC-MS can be considered as a reference method. Repeatability and reproducibility are mostly applied as indicators of method precision. Lot to lot reproducibility and shelf-life stability can influence accuracy and precision of the method.

Other parameters that should be noticed in developing a practical method for mycotoxin include portability for on-site applications, user-friendliness, and low-cost detection.

According to the above-mentioned factors, some of the researches reported in this review have the potential to be industrialized. Most of enzymatic detection methods based on DNAzyme, reported in Table 1, show high sensitivity and selectivity and good precision which make them suitable for mycotoxin detection. However, long incubation time, multi-step washing, and complex operation are considered as main limitations toward their practical applications. Although reported AchE-based assay is simple, rapid, and low-cost, it cannot be considered as a very selective method due to the presence of other analytes with AchE inhibitory action such as pesticides and so on.

Reported ELISA-based methods (Table 2) with high sensitivity and selectivity can be ideal for industrialization after a few modifications related to the detection time. In terms of meeting current trends, multiplex ELISA demonstrated by Urusov et al. [64] for simultaneous detection of AFB1, OTA, and ZEN with a high detectable signal and high sensitivity is an ideal platform for practical multiplex analysis. The proposed assay was successfully validated for food samples with complex matrices. Multiplex nanoarray based on ELISA technique developed by McNamee et al. [65] was able to detect ZEA, T-2 toxin, and FMB1 in a simple way with high sensitivity and accuracy. The established protocol offered a higher throughput of samples and potential feasibility for easy to use and multiplex detection compared to the other developed ELISA methods.

Developed ELISA protocols based on nanomaterials as enzyme substitute can be also considered for practical applications due to greater stability than conventional ELISA. In this regard, the nanozyme-linked immunosorbent assay based on MOFs (Table 2) for AFB1 detection is a good example [63]. The developed method showed high sensitivity and selectivity, high accuracy and excellent stability without false positive and false negative results. Plasmonic ELISAs using enzyme and nanomaterials can be suitable for naked-eye detection and on-site application with no need for ELISA reader devices. Quantitative results can be obtained with smartphone-based signal readout systems. In this case, plasmonic ELISA methods demonstrated by Xiong et al. [66] and Pei et al. [60] can be mentioned.

LFIAs are strong competitors on the market for mycotoxin detection due to their unique features such as high sensitivity and selectivity, low-cost, short detection time, portability, and user-friendliness [29]. Therefore, research and development in the field of these popular detection kits is very important. Many efforts have been made for the development of quantitative and multiplex LFIA test strips. In this regard, LFIA based on AuNPs and TRFMs for multiplex detection of AFB1, ZEN, FMB1, DON, and T-2 toxin along with a smartphone-based quantitative dual detection mode device [30] is a good example of a multiplex and quantitative analysis (Table 3). Developed strips showed high sensitivity and reliability. Application of smartphone provided a low-cost and portable quantitative method. Stability of strips during storage is a main parameter in practical application which should be checked in this study.

Aptamer-based LFAs can be suitable alternatives for antibody-based LFA in the future due to lower cost and higher stability of aptamers compared to antibodies. For example, aptamer-based LFA developed by Wu et al. [75] exhibited high sensitivity and high stability (2 months at room temperature), and short detection time (5 min) for the determination of ZEN. Another example is aptamer-based LFA for OTA detection with high sensitivity and excellent selectivity [76]. It was stable for 6 months at room temperature.

Only a few studies are available in the field of microfluidic-based assays for mycotoxin detection. However, this method could have a good future for mycotoxins detection. Among developed microfluidic methods in mycotoxin analysis, paper-based systems show greater potential for commercialization due to simplicity, low-cost and portability. They do not need additional equipment such as pump to generate flow. On the other hand, quantification of the results can be made with a smartphone. As reported in Table 4, the proposed µPAD by Kasoju et al. [88] for AFB1 detection showed high sensitivity, low-cost, short detection time (>1 min), and portability. Moreover, the proposed µPAD for DON detection has a great potential for industrialization [90].

## 5. Conclusions and Future Directions

Development of a suitable biosensor or bioassay for mycotoxin analysis as alternative approaches to conventional sophisticated techniques such as chromatography-based methods is of great importance in the field of biosensing research. Among different sensing strategies, colorimetric approaches are very popular, simple, and convenient and present great value for on-site detection. Colorimetric methods are categorized based on type of colored label and the medium in which the reaction develops (solution-based and solid substrate-based). ELISA and LFA are considered as the most common solution-based and solid-substrate-based colorimetric detection methods due to their unique features. In addition to a wide variety of researches in these areas, multiple companies worldwide are developing and producing detection kits relying on these two techniques. The products from different suppliers can be different in terms of sensitivity, selectivity, the type of matrix used, detection time, and number of detectable mycotoxins.

Notwithstanding the great success and advance in this field, hand-held digital biosensors and smartphone-based quantitative detection are desired for the future market, aiming for an intuitive user experience. Furthermore, because of the very competitive market for mycotoxin test kits, the future market welcomes products capable of performing multiplex analysis and high portability.

It is also possible that new recognition elements, such as aptamer and molecularly imprinted polymer (MIP), will replace the antibody commercially available tests for a more convenient future technology. Despite their merits, aptasensing assays remain relatively immature for industrial monitoring. This is probably due to the short history of reproducible aptasensors in real sample analysis and the lack of broad dissemination of results on the market saturated with immune kits. Nevertheless, owing to the advantages and prospects offered by the mass production of specific aptamers, its contribution to the colorimetric sensors market in food safety is expected to evolve rapidly and shape the future market.

## Figures and Tables

**Table 1:** Representative examples of recent developed enzyme-based probes for the colorimetric detection of mycotoxins.

Strategy	Detection Probe	Target	LOD	Linear Range	Specificity	Sample	Advantages/Disadvantages	Ref.
Colorimetric aptasensor based on HRP-encapsulated liposome which catalyzed TMB oxidation	HRP	OTA	0.023 ng·mL^−1^	0.05–2.0 ng·mL^−1^	High	Corn	Simple, low-cost, highly selective, sensitive and reliable/long detection time (60 min)	[11]
Combination of an ingenious hairpin DNA probe with exonuclease III (Exo III)-assisted signal amplification and TMB oxidation	DNAzyme	AFB1	1 pM	1 pM–100 nM	High	Peanut	High sensitivity, good selectivity, simple operation, wash-free, label-free format, low-cost, applicability to samples with complex matrices/long incubation time (40 min)	[15]
Dual aptamer-DNAzyme in combination with apta-magnetic separation	DNAzyme	AFB1	22.6 ppb	0–200 ppb	Good	Corn, rice, groundnut, black pepper, chili	High sensitivity, good selectivity, low-cost, reliable/long incubation time of DNAzyme (30 min)	[17]
Detection aptamer containing a DNAzyme sequence and an RCA priming sequence for the isothermal DNA amplification	DNAzyme	OTA	1.09 × 10^−12^ ng·mL^−1^	10^−12^–10 ng·mL^−1^	High	Urine	High sensitivity and selectivity, applicability to biological samples with complex matrices/long incubation time and complicated operation with multiple steps of washing and separation	[16]
Combination of DNA aptamer and two split hemin-binding DNAzyme halves and G-quadruplex formation of a split DNAzyme-hemin/aptamer complex with peroxidase mimicking activity in the absence of AFB1	DNAzyme	AFB1	0.1 ng·mL^−1^	0.1–10^4^ ng·mL^−1^	High	Corn	High sensitivity and selectivity simple and low-cost/long incubation time (70 min)	[21]
Inhibition of chitosan-immobilized AchE activity by AFB1and Ellman’s method	AchE	AFB1	Not reported	Not reported	Good	Corn	Simple, rapid (detection time ≈ 8 min), low-cost, portable/failure to report LOD and linear range	[19]

**Table 2:** Representative examples of recent developed nanomaterial-based methods for the colorimetric detection of mycotoxins.

Strategy	Detection Probe	Target	LOD	Linear Range	Specificity	Sample	Advantages/Disadvantages	Ref.
Aptamer assay based on AuNPs aggregation by poly diallyldimethyl ammonium chloride polymer (PDDA).	AuNPs	OTA	0.009 ng·mL^−1^	0.05–50 ng·mL^−1^	High	Chinese liquor sample	Cost-effectiveness, few steps, rapid detection (15 min), good sensitivity/Possible cross-reactivity at high target concentrations.	[38]
AuNP dimer disassembly by the target-induced release of complementary DNA probes.	AuNPs	OTA	0.05 nM	0.2–250 nM	High	Red wine	Improved sensitivity, low-cost, short detection time (15 min)/Difficult applicability to colored complex samples	[39]
Double calibration curve of label free aptasensing assay based on salt-induced aggregation of AuNPs.	AuNPs	OTA	0.03 ng·mL^−1^	0.03–316 ng·mL^−1^	Good	Corn	Widened detection range, enhanced sensitivity, reliability, rapid detection/low selectivity	[40]
Peroxidase-like activity of AuNPs in the presence of H_2_O_2_ and TMS substrate.	AuNPs	ZEN	10 ng·mL^−1^	10–250 ng·mL^−1^	High	Corn and corn oil	Simple one-step assay, short detection time/Relatively high detection limit	[41]
Chemical nano-sensor based on cysteamine-modified AuNPs aggregation via electrostatic interaction with hydrolyzed target.	AuNPs	FB1	0.90 μg·kg^−1^	2–8 μg·kg^−1^	Low	Corn	Simple one-step assay, Rapid homogenous test (3 min)/Real sample interferences, low sensitivity and selectivity.	[42]
ALP- induced gold nanoparticle aggregation mediated by MnO_2_ nanosheets reduction in the presence of generated ascorbic acid.	AuNPs, MnO_2_ nanosheets	OTA	5.0 nM	6.25–750 nM	High	Grape juice & red wine	Enzymatic amplification, high selectivity/multi reaction steps, possible cross reactivity in real samples	[37]
Colorimetric aflatoxins immunoassay by using mesoporous silica nanoparticles decorated with gold nanoparticles.	AuNPs@m-SiNPs nanocomposite	AFs (AFB1, AFB2, and AFG2)	0.16 ng·mL^−1^	1–75 ng·mL^−1^ for AFB1	High	Nuts, cornflakes, cornmeal, peanuts, peanut butter and pecan nuts	High sensitivity, versatile real matrix applicability, 30 min incubation time/long synthesis and modification of transducer	[43]
AFB1 hydrolyzed to phenolate anions react with curcumin enol form-Zn red complex to give curcumin enol form-ZnO-Phenol yellow complex.	ZnO NPs	AFB1	11 µg·kg^−1^	0–36 µg·kg^−1^	Good	Rice	Simple and rapid detection, bioreceptor-free sensor, HPLC validation/Chemical modification of target	[44]
Cascade aptasensor by double catalytic amplifications using ALP activity combined to the inhibition of the MnO_2_ oxidase-mimicking activity.	MnO_2_ nanosheets	OTA	0.07 nM	1.25–250 nM	Excellent	Grape juice	Amplified colorimetric signal, high sensitivity and selectivity/Many washing and addition steps	[34]
Simultaneous dual target detection via the combination of two the catalysis of TMB under acidic conditions and the release of TP under alkaline conditions.	Fe_3_O_4_-GO nanocomposite and AuNPs	AFB1	1.5 ng·mL^−1^	5–250 ng·mL^−1^	High	Peanuts	Multiplexed detection, high sensitivity and selectivity/Tedious probes synthesis, specific pH and temperature conditions, long incubation time (90 min), multiple steps of washing and separation	[45]
		OTA	0.15 ng·mL^−1^	0.5–80 ng·mL^−1^				
Salt-induced coagulation of iron-modified 2D covalent triazine framework nanosheets (2D Fe-CTFs) that showed strong peroxidase-like activity	2D Fe-CTFs	OTA	NR	0.2–0.8 μM	NR	NR	Promising proof of concept/limited detection range, analytical performances not reported	[46]
PAD sensor array based on silver and gold nanoparticles aggregation synthesized by three different capping agents.	AgNPs and AuNPs	AFB1	2.7 ng·mL^−1^	3.1 ng·mL^−1^ –7.8 μg·mL^−1^	Excellent	Mixtures of pistachio, wheat and coffee, milk	Very fast colorimetric response (5 s), multiplexed detection of five mycotoxins, low cost, device portability/Optical nanoprobes fabrication	[47]
		AFG1	7.3 ng·mL^−1^	8.2 ng·mL^−1^–8.4 μg.mL^−1^				
		AFM1	2.1 ng·mL^−1^	2.5 ng·mL^−1^–8.2 μg.mL−1				
		OTA	3.3 ng·mL^−1^	4.0 ng·mL^−1^–3.8 μg.mL^−1^				
		ZEN	7.0 ng·mL^−1^	8.0 ng·mL^−1^–7.9 μg.mL^−1^				

**Table 3:** Representative examples of recent developed ELISA methods for the detection of mycotoxins.

Strategy	Target	LOD	Linear Range	Specificity	Sample	Advantages/Disadvantages	Ref.
Colorimetric ELISA based on glucose oxidase-regulated the color of bromocresol purple acid-base indicator	AFB1	Cutoff limit: 100 pg·mL^−1^	25–200 pg·mL^−1^	Excellent	Corn	High sensitivity and selectivity, good repeatability/long incubation time, multi-step washing	[58]
Plasmonic ELISA based on the urease-induced metallization of gold nanoflowers	OTA	8.205 pg·mL^−1^	5.0–640 pg·mL^−1^	Excellent	Rice, corn, wheat, white wine	High sensitivity and selectivity, robust, and high-throughput, good repeatability/long incubation time, multi-step washing	[60]
DLS-ELISA based on AuNPs aggregation via hydroxyl radicals produced by HRP activity on H_2_O_2_	AFB1	0.12 pg·mL^−1^	Not reported	Excellent	Corn	High sensitivity and selectivity, reliable, good repeatability, high accuracy/long incubation time, multi-step washing	[61]
Apta-ELISA based on target capture by OTA specific aptamer and color development by anti-rabbit secondary antibody labeled with ALP	OTA	0.84 pg·mL^−1^	1 pg·mL^−1^–1 µg·mL^−1^	Good	Groundnut, coffee bean	High sensitivity, low-cost/long incubation time, multi-step washing, cross-reactivity, matrix interference	[62]
A nanozyme-linked immunosorbent assay based on metal–organic frameworks (MOFs) instead of enzyme to catalyze chromogenic TMB	AFB1	0.009 ng·mL^−1^	0.01–20 ng·mL^−1^	Excellent	Peanut milk, soymilk	High sensitivity and selectivity, high catalytic activity and stability of MOF, good recovery rate and accuracy, avoiding false positive and false negative results/long incubation time, multi-step washing	[63]
Multiplexed ELISA based on immobilization of protein-analyte conjugates in separate wells	OTA	4.0 ng·mL^−1^	4.0–120 ng·mL^−1^	Not reported	Poultry, corn	High sensitivity, reduction of simultaneous detection time of three mycotoxins/long incubation time, multi-step washing	[64]
	AFB1	0.1 ng·mL^−1^	0.1–1.0 ng·mL^−1^				
	ZEN	0.3 ng·mL^−1^	0.3–50.0 ng·mL^−1^				
Multiplex nanoarray based on ELISA technique via nano-spotting of mycotoxin-protein conjugates into single wells of a microplate	ZEN T-2 toxin FB1	IC50: 197.4 0.7 216.7 µg·kg^−1^ in wheat	Not reported	High	Wheat, corn	High sensitivity and selectivity, reduction of simultaneous detection time of three mycotoxins, throughput, easily adaptable by end users/long incubation time, multi-step washing	[65]
Plasmonic ELISA based on the HRO-assisted etching of gold nanorods	AFB1	Cutoff limit: 12.5 pg·mL^−1^	3.1–150 pg·mL^−1^	Excellent	Corn	High sensitivity and selectivity, high accuracy and precision, portable, equipment-free/long incubation time, multi-step washing	[66]

**Table 4:** Representative examples of recent developed LFA strip tests for the detection of mycotoxins.

Strategy	Target	LOD	Linear Range	Specificity	Sample	Advantages/Disadvantages	Ref.
Dual color AuNPs and a single test line for simultaneous detection of two mycotoxins	AFB1 FBs	0.5 ng·mL^−1^ 20 ng·mL^−1^	Not reported	Not reported	Wheat, pasta	High sensitivity, simple, low-cost, rapid (detection time of 10 min), semi-quantitative, reliable/no evaluation of selectivity and stability	[71]
Magneto-gold nanohybrid based LFA for simultaneous separation and target detection	OTA	0.094 ng·mL^−1^	0.098–12.5 ng·mL^−1^	High	Grape juice	High sensitivity and selectivity, simple, low-cost, rapid (detection time of 15 min), semi-quantitative, high precision in complex matrices, high reproducibility/no evaluation of stability	[72]
AuNPs and TRFMs-based lateral flow immunoassays for multiplex detection mycotoxins along with a smartphone-based quantitative dual detection mode device	AFB1 ZEN Deoxynivalenol T-2 toxin FB1	AuNPs-LFA: 0.59 0.24 0.32 0.90 0.27 ng·mL^−1^ TRFMs-LFA: 0.42 0.10 0.05 0.75 0.04 ng·mL^−1^	Not reported	Low	Maize, wheat, bran	High sensitivity, simple, low-cost, rapid, reliable, quantitative, portable/no evaluation of stability, high cross reactivity with other mycotoxin in a single class	[30]
Application of GO and carboxylated GO instead of AuNPs as label	AFB1	0.3 ng·mL^−1^	Not reported	Not reported	Peanut oil, maize, rice	High sensitivity, high stability (4 months), simple, low-cost, rapid, reliable, rapid (detection time of 15 min), acceptable reproducibility/qualitative, no evaluation of specificity	[31]
Aptamer-based competitive LFA	ZEN	vLOD: 20 ng·mL^−1^ qLOD: 5 ng·mL^−1^	5–200 ng·mL^−1^	High	Corn	High sensitivity and selectivity, high stability (2 months at room temperature), simple, low-cost, short detection time (5 min), portable/no evaluation of reproducibility	[75]
AuNPs-aptamer conjugate as recognition element and label; immobilization of biotinylated DNA probe 1 and probe 2 on test and control lines, respectively	OTA	1 ng·mL^−1^	Not reported	Excellent	*Astragalus membranaceus*	High sensitivity and selectivity, cost-effective, robust, high stability (6 months at room temperature), simple, short detection time (15 min), portable/no evaluation of reproducibility, qualitative	[76]
Design a smart analysis platform for multiplex LFIA based on AuNPs as label and five test lines	AFB1 ZEN Deoxynivalenol T-2 toxin FB1	4 40 200 10 20 µg·kg^−1^	Not reported	Low	Wheat	High sensitivity, cost-effective, robust, simple, short detection time (15 min), portable, quantitative/no evaluation of reproducibility and stability, high cross reactivity with other mycotoxin in a single class	[77]
Enhanced signal sensitivity in LFIA using multi-branched gold nanoflowers	AFB1	0.32 pg·mL^−1^ in rice	0.5–25 pg·mL^−1^	Low	Rice	Excellent sensitivity, cost-effective, simple, short detection time (15 min), portable, quantitative/no evaluation of reproducibility and stability, high cross reactivity with AFG2	[78]
Multiplex LFIA based on AuNPs as label	AFB1 ZEN OTA	0.1–0.13 0.42–0.46 0.19–0.24 µg·kg^−1^	Not reported	Not reported	Corn, rice, peanut	High sensitivity, cost-effective, simple, short detection time (15 min), portable, quantitative/no evaluation of reproducibility, selectivity and stability	[79]
AuNPs-based LFIA with silver staining for signal amplification	FB1 Deoxynivalenol	cut-off values: 2.0 40 ng·mL^−1^	Not reported	Not reported	Maize	High sensitivity, low-cost, simple, short detection time, portable/no evaluation of reproducibility, selectivity and stability	[80]
LFIA based on multifunctional photothermal contrast Fe_3_O_4_@Au supraparticle (Fe_3_O_4_@Au SP)	OTA	0.12 pg·mL^−1^	1 pg·mL^−1^–1 µg·mL^−1^	Good	Corn, peanut, soybean	High sensitivity and selectivity, low-cost, simple, reliable/no evaluation of reproducibility and stability	[81]
A LFIA using Prussian blue nanoparticle (PBNP) as a peroxidase mimicking label for TMP catalysis	OTA	10 pg·mL^−1^	10 pg·mL^−1^–1 µg·mL^−1^	High	Human serum	High sensitivity and selectivity, low-cost, simple, reliable/no evaluation of reproducibility and stability	[32]

**Table 5:** Representative examples of recent developed microfluidic-based assays for the detection of mycotoxins.

Strategy	Target	LOD	Linear Range	Specificity	Sample	Advantages/Disadvantages	Ref.
PDMS-based microfluidic immunoassay using antibody labeled with HRP	OTA AFB1 ZEN	<40 0.1–0.2 <10 ng·mL^−1^	−	High	Corn	Good sensitivity, high selectivity, low-cost, short detection time (10 min), portable/no evaluation of reproducibility and stability, lots of user-intervention steps, semi-quantitative	[83]
Microfluidic immunoassay based on combination target binding to AuNPs-mAb and immunogold amplification of AuNPs-mAbs-AME	Altenariol monomethyl ether	12.5 pg·mL^−1^ 200 pg·mL^−1^	12.5–200 pg·mL^−1^ 200–1000 pg·mL^−1^	High	Apple, cherry, orange	High sensitivity and selectivity, low-cost, short detection time (15 min), portable, simultaneous analysis of six samples, quantitative/no evaluation of reproducibility and stability	[87]
Development of a µPAD based on salt-induced aggregation of AuNPs in the presence of analyte	AFB1	10 nM	1 pM–1 µM	High	Water	High sensitivity and selectivity, cost-effective, short detection time (> 1 min), portable/no evaluation of reproducibility and stability, no evaluation of food matrix, qualitative	[88]
Colorimetric competitive immunoassay into a paper microfluidic device using AuNPs as signal indicator	Deoxynivalenol	0.644 ng·mL^−1^	0.01–20 ng·mL^−1^	High	Wheat, corn	High sensitivity and selectivity, rapid (detection time of 12 min), low-cost, portable, and reliable/qualitative, no evaluation of reproducibility and stability	[90]

**Table 6:** Main companies providing commercial colorimetric immuno-kits for mycotoxins analysis.

Company	Kit	Type of Detection	Mycotoxins						Time (min)	Multiplexing	Ref.
			AFs	OTA	FUM	ZEN	DON	T2/HT2			
Astori Tecnica	ELISA	QNT, Semi-QNT for OTA	✓	✓	✓	✓	✓	✓	20–30	No	[95]
	LFIA	QLT, QNT	AFM1	(−)	(−)	(−)	(−)	(−)	10		
Charm Sciences Inc.	LFIA	QNT	✓	✓	✓	✓	✓	✓	3–5	No	[96]
CUSABIO	ELISA	QNT	AFB1	✓	(−)	✓	✓	T2	20	No	[97]
	LFIA	QNT	AFB1	✓	(−)	✓	✓	T2	3–5		
Elabscience	ELISA	QNT	✓	✓	✓	✓	✓	✓	75	No	[98]
	LFIA	QNT	✓	✓	✓	✓	✓	✓	8		
EnviroLogix	LFIA	QLT, QNT	✓	✓	✓	✓	✓	✓	2–4	Yes (AF, ZEN, DON, FUM)	[99]
Eurofins	ELISA	QNT	✓	✓	✓	✓	✓	✓	15–75	No	[100]
	LFIA	QNT	✓	(−)	✓	✓	✓	(−)	5		
Helica	ELISA	QNT	✓	✓	✓	✓	✓	(−)	NR	No	[101]
Neogen	ELISA	QNT	✓	✓	✓	✓	✓	✓	10–20	No	[102]
	LFIA	QLT, QNT	✓	✓	✓	✓	✓	✓	3–8		
R-Biopharm	ELISA	QNT	✓	✓	✓	✓	✓	✓	45–150	No	[103]
	LFIA	Semi-QNT, QNT	✓	(−)	✓	✓	✓	✓	5		
Romer Labs	ELISA	QNT	✓	✓	✓	✓	✓	✓	15	Yes (up to 6 mycotoxins)	[104]
	LFIA	QLT, QNT	✓	✓	✓	✓	✓	(−)	3	No	
Vicam	LFIA	Semi-QNT, QNT	✓	✓	✓	✓	✓	✓	5	No	[105]
Beacon Analytical Systems	ELISA	QNT	✓	✓	✓	✓	✓	✓	15–75	No	[106]
Creative Diagnostics	ELISA	QNT	✓	✓	✓	(−)	✓	✓	15–120	No	[107]
	LFIA	QLT, Semi-QNT, QNT	✓	✓	✓	✓	✓	✓	3–10		
PerkinElmer	ELISA	QNT	✓	✓	✓	✓	✓	✓	15–60	No	[108]
	LFIA	QNT	✓	✓	✓	✓	✓	✓	4–6		
Unisensor	LFIA	QNT	✓	(−)	(−)	(−)	(−)	(−)	10	No	[109]
Pribolab	ELISA	QNT	✓	✓	✓	✓	✓	✓	NR	No	[110]
	LFIA	QLT, QNT	✓	✓	✓	✓	✓	(−)	10–12		
Randox	ELISA	QNT	✓	(−)	(−)	(−)	(−)	(−)	NR	No	[111]
	Biochip Arrays	QNT	✓	✓	✓	✓	✓	✓	120	Yes (up to 10 mycotoxins)	
Novakits	ELISA	Semi-QNT, QNT	✓	✓	✓	✓	✓	✓	15–70	No	[112]
	LFIA	QNT	✓	✓	✓	✓	✓	✓	5	Yes (7 mycotoxins)	
Sigma	ELISA	QNT	✓	✓	✓	✓	✓	(−)	NR	No	[113]
Bio-Check	ELISA	QNT	✓	✓	✓	✓	✓	(−)	NR	No	[114]
	LFIA	QNT	✓	✓	✓	(−)	✓	(−)	3–5		

QNT = Quantitative; Semi-QNT = Semi-quantitative; QLT = Qualitative; NR = Not reported; ✓ = Available detection kit for the specific mycotoxin, (−) = Not available.
